# Final Results of the ILLUMINATE-A Phase 3 Clinical Trial of Lumasiran for Primary Hyperoxaluria 1

**DOI:** 10.2215/CJN.0000000916

**Published:** 2025-12-04

**Authors:** Yaacov Frishberg, Jeffrey M. Saland, John C. Lieske, Weiming Du, Martin Coenen, Julien Hogan, Anne-Laure Sellier-Leclerc, Jaap W. Groothoff, Cristin Kaspar, John M. Gansner, Sally-Anne Hulton

**Affiliations:** 1 Division of Pediatric Nephrology, Shaare Zedek Medical Center, Jerusalem, Israel and Faculty of Medicine, Hebrew University, Jerusalem, Israel; 2 Icahn School of Medicine at Mount Sinai, New York, New York; 3 Division of Nephrology and Hypertension, Mayo Clinic, Rochester, Minnesota; 4 Alnylam Pharmaceuticals, Cambridge, Massachusetts; 5 Institute of Clinical Chemistry and Clinical Pharmacology, University Hospital Bonn, Bonn, Germany; 6 Pediatric Nephrology Department, Hôpital Robert-Debré, APHP, Centre Référence Maladie Rares MARHEA, ERKnet, Paris, France; 7 Hôpital Femme Mère Enfant en Centre d'Investigation Clinique, Institut National de la Santé et de la Recherche Médicale (INSERM), Hospices Civils de Lyon, ERKnet, Bron, France; 8 Department of Pediatric Nephrology, Emma Children's Hospital, Amsterdam UMC, University of Amsterdam, The Netherlands; 9 Department of Nephrology, Birmingham Women's and Children's Hospital, Birmingham, United Kingdom

**Keywords:** CKD, chronic nephropathy, clinical trial, kidney stones, renal function decline, genetic diseases and development

## Abstract

**Key Points:**

- Primary hyperoxaluria type 1 is a genetic disorder associated with hepatic oxalate overproduction, kidney failure, and systemic oxalosis.
- Lumasiran treatment for up to 60 months was associated with sustained reductions in urinary and plasma oxalate and encouraging clinical outcomes.
- Lumasiran's safety profile was acceptable with mild injection site reactions being the most common adverse events.

**Background:**

Primary hyperoxaluria type 1 (PH1) is characterized by hepatic oxalate overproduction, kidney stones, and progressive kidney disease. Lumasiran is the first approved treatment for PH1.

**Methods:**

ILLUMINATE-A (NCT03681184) was a 60-month pivotal, phase 3, multinational clinical trial of lumasiran which consisted of a 6-month double-blind, placebo-controlled period (6M DB), followed by a treatment extension period of up to 54 months in which all patients received lumasiran. Eligible patients were aged ≥6 years with genetically confirmed PH1, eGFR ≥30 ml/min per 1.73 m^2^, and mean 24-hour urinary oxalate (UOx) excretion ≥0.70 mmol/24 h per 1.73 m^2^.

**Results:**

Of 39 patients enrolled, 24 of 26 randomized to lumasiran in the 6M DB (lumasiran/lumasiran group) and 13 of 13 randomized to placebo in the 6M DB (placebo/lumasiran group) completed the study. Sustained reductions in 24-hour UOx were observed in both treatment groups. At month 60 relative to study baseline, the mean (SEM) 24-hour UOx percentage reductions were 54 (6) and 54% (8%) in the lumasiran/lumasiran and placebo/lumasiran groups, respectively, and the mean (SEM) plasma oxalate concentrations had decreased by 35 (5) and 38% (7%). eGFR remained stable through the end of the study in both treatment groups. Kidney stone event rates during the study were 0.47 (95% confidence interval, 0.36 to 0.62) and 0.54 (0.37 to 0.78) per patient-year in the lumasiran/lumasiran group and placebo/lumasiran group, respectively. Medullary nephrocalcinosis grade improved at the end of the study in 21 of 28 (75%) patients with nephrocalcinosis at baseline and an end of study assessment. The safety profile of lumasiran was acceptable. Injection site reactions were the most common adverse events during lumasiran treatment. Most adverse events were mild or moderate in severity.

**Conclusions:**

Lumasiran treatment for up to 60 months in ILLUMINATE-A was associated with sustained reductions in UOx excretion and plasma oxalate concentration, encouraging clinical outcomes including stable eGFR in a population that would be expected to show eGFR decline, reduced kidney stone event rates, improved medullary nephrocalcinosis, and indications of improved health-related quality of life.

Clinical Trial registry name and registration number: ClinicalTrials.gov NCT03681184.

**Podcast:**

This article contains a podcast at https://dts.podtrac.com/redirect.mp3/www.asn-online.org/media/podcast/CJASN/2026_03_18_CJASNMar.21.3.31820.mp3

## Visual Abstract

**Figure:**
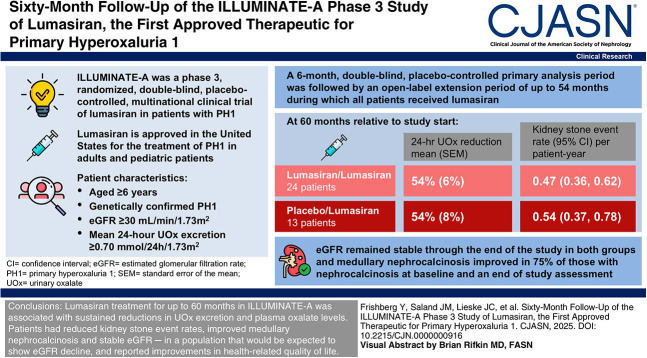

## Introduction

Primary hyperoxaluria type 1 (PH1) is a rare recessive disorder characterized by hepatic oxalate overproduction, increased kidney oxalate excretion, and calcium oxalate crystal formation in the kidneys and urinary tract.^1^ Patients may experience nephrolithiasis and/or nephrocalcinosis, which can ultimately progress to kidney failure and systemic oxalosis.^1,2^ PH1 symptoms and treatments before the introduction of ribonucleic acid interference (RNAi) therapies have a substantial negative effect on quality of life for patients and caregivers.^3,4^

The primary therapeutic aim in PH1 is preservation of kidney function. Historically, nontransplant PH1 treatments have included hyperhydration, crystallization inhibitors, pyridoxine, and, in severe cases, intensive dialysis. Lumasiran, an RNAi therapeutic, targets and promotes degradation of the messenger RNA encoding glycolate oxidase in hepatocytes, reducing hepatic oxalate production.^5^ Lumasiran is approved for the treatment of PH1 in pediatric and adult patients in multiple regions, including the United States, European Union, the United Kingdom, Canada, Australia, and several countries in Latin America and the Middle East.^5–7^ More recently, nedosiran, an RNAi therapeutic targeting the messenger RNA encoding lactate dehydrogenase A in hepatocytes, has become available in the United States for the treatment of PH1 in children aged 2 years and older with relatively preserved kidney function.^8^

Lumasiran was investigated in several phase 3 clinical trials in a diverse population of patients with PH1, including ILLUMINATE-A,^9,10^ ILLUMINATE-B,^11,12^ and ILLUMINATE-C.^13,14^ ILLUMINATE-A was a randomized, double-blind (DB), placebo-controlled, multinational trial (ClinicalTrials.gov: NCT03681184; EudraCT: 2018-001981-40) in patients aged 6 years or older with genetically confirmed PH1 (*i.e*., with mutation of the *AGXT* gene, which encodes alanine:glyoxylate aminotransferase^15^) and an eGFR ≥30 ml/min per 1.73 m^2^. During a 6-month DB phase, lumasiran treatment was associated with significant reductions in urinary oxalate (UOx) and plasma oxalate (POx).^9^ An open-label extension period of up to 54 months followed the DB period, allowing for up to 60 months of lumasiran treatment in the lumasiran/lumasiran group or up to 54 months of lumasiran treatment in the placebo/lumasiran group.^10^ The results of an interim analysis through Month 36 were reported previously.^10^ Here, we report data from the final, 60-month analysis of the ILLUMINATE-A trial.

## Methods

### Study Design and Patients

The study design has been described in detail.^9^ In brief, ILLUMINATE-A was a pivotal, multinational trial conducted from January 2019 (start of enrollment) to January 2024 at 16 study sites (Europe, the Middle East, and North America). Eligible patients were aged 6 years or older with genetically confirmed PH1, eGFR ≥30 ml/min per 1.73 m^2^, and mean 24-hour UOx excretion ≥0.70 mmol/24 h per 1.73 m^2^ from the first two valid 24-hour urine collections. Patients taking pyridoxine for the treatment of PH1 were required to have been on a stable regimen for ≥90 days before randomization and willing to remain on this regimen for 12 months from first study drug administration.

This 60-month study included an initial 6-month, DB, randomized, placebo-controlled period in which patients were randomized to either placebo or lumasiran, followed by a 3-month blinded treatment extension period and a 51-month open-label extension period in which all patients received lumasiran. Patients randomized to lumasiran in the initial 6-month randomized, placebo-controlled period are referred to as the lumasiran/lumasiran group. Patients randomized to placebo in the initial 6-month randomized, placebo-controlled period are referred to as the placebo/lumasiran group. Lumasiran 3 mg/kg was administered as a loading dose once monthly for three doses, followed by a maintenance dose every 3 months beginning 1 month after the last loading dose.

The study was approved by central and local institutional review boards or ethics committees and conducted in accordance with Good Clinical Practice guidelines and the provisions of the Declaration of Helsinki.^16^ Patients or their legal guardians provided written informed consent.

### Outcome Measures

Urine and blood samples were collected for assessment of oxalate and glycolate concentrations, which were measured using liquid chromatography-tandem mass spectrometry.^17^ Blood samples were collected for assessment of serum creatinine, which was used for calculation of eGFR. The eGFR values at each study time point were calculated using the patient's age at the time of blood sampling, applying the Modification of Diet in Renal Disease formula for patients aged ≥18 years at the time of screening and the Schwartz Bedside Formula for patients aged <18 years at the time of screening.^18,19^

A kidney stone event (KSE) was defined as a visit to a health care provider (outpatient clinic, urgent care, emergency department, or procedure) because of a kidney stone, medication for kidney colic, stone passage, and/or macroscopic hematuria due to a kidney stone. KSE rates were calculated as the total number of KSEs divided by the total patient exposure time (events per patient-year [PY]). Medullary nephrocalcinosis grade was assessed using kidney ultrasound and an established grading scale with excellent or “almost perfect” intraobserver and interobserver agreement.^20,21^ Higher grades (range: 0–3) indicate greater severity.^20^ Overall changes in nephrocalcinosis grade relative to baseline (BL), accounting for both kidneys, were expressed categorically as no change, improving, worsening, and indeterminate (one kidney improving and one kidney worsening). Kidney ultrasounds were reviewed centrally by radiologists who were not investigators or site personnel and who were blinded to treatment and visit.

Health-related quality of life (HRQOL) questionnaires were used to evaluate overall health status. Patient age at screening determined which age-specific questionnaires were used for the duration of the study. Assistance with completing questionnaires was provided by the site if requested by the patient. The EQ-5D 5 level questionnaire (EQ-5D-5L) and EQ-5D questionnaire for Y (EQ-5D-Y) consist of five domains and a visual analog scale (VAS); higher scores indicate better health status.^22^ The EQ-5D-5L was completed by patients aged ≥18 years at screening, and the EQ-5D-Y was completed by patients aged <18 years at screening. The Kidney Disease Quality of Life Questionnaire (KDQOL; completed by patients aged ≥18 years at screening) and Pediatric Quality of Life Inventory (PedsQL; completed by patients aged <18 years at screening) subscales and summary scores assess self-reported HRQOL, with higher scores indicating better health status.^23,24^ KDQOL subscales include Short Form (SF)-12 (physical component summary and mental component summary),^25^ effects of kidney disease, burden of kidney disease, and symptoms/problems.

Safety assessments included frequency and seriousness of adverse events (AEs) during the extension period. Blood samples were collected to evaluate antidrug antibodies (ADAs) using a validated enzyme-linked immunoassay.

### End Points

The primary end point of the randomized, controlled phase of the trial was percentage change in 24-hour UOx excretion corrected for body surface area from BL (defined as the median of all valid 24-hour urine assessments collected before the first dose date/time of study drug [lumasiran or placebo] without any non–protocol-related sample issues) to the Month 6 time point (defined as the average of values from Month 3 to Month 6). Secondary end points included percentage change in 24-hour UOx excretion during the extension period, absolute changes in 24-hour UOx excretion from BL to Month 6 and during the extension period, percentage and absolute changes in POx from BL (defined as the mean of all POx measurements before the first dose date/time of study drug [lumasiran or placebo] in the 6-month DB period) to Month 6, and changes in eGFR from BL (defined as the last eGFR assessment before the first dose of study drug in the 6-month DB period) to Month 6 and during the extension period. Exploratory end points included changes in UOx:creatinine (UOx:Cr) ratio as assessed in random spot urine collections, plasma glycolate, KSE rate, nephrocalcinosis, EQ-5D and EQ-5D VAS, KDQOL, and PedsQL (generic and ESKD modules) from BL to Month 6 and during the extension period. BLs for spot UOx:Cr and plasma glycolate were defined as the mean of all measurements before the first dose date/time of study drug (lumasiran or placebo) in the 6-month DB period. BLs for nephrocalcinosis and HRQOL measures were defined as the last assessments before the first dose of study drug (lumasiran or placebo) in the 6-month DB period.

### Statistical Analyses

All end points were summarized descriptively during the extension period. Most analyses were based on the full analysis set, defined as all randomized patients who received any amount of study drug (*i.e*., placebo or lumasiran). Analyses of POx levels over time included patients who received any amount of study drug and had a BL POx level ≥1.5 times the lower limit of quantification (5.55 *µ*mol/L). Safety analyses were based on the all-lumasiran-treated set, defined as all patients who received any doses of lumasiran, including patients who took lumasiran during the 6-month DB period and patients who initially took placebo during the DB period and then switched to lumasiran during the extension period.

Hyperhydration was defined as a specific order by a health care provider to ingest large volumes of fluid. BL hyperhydration status was the latest status before the first dose of study drug (lumasiran or placebo) during the DB period. In the study, patients could have multiple changes in hyperhydration status, but only the first change that occurred during the study is summarized.

Available long-term safety data, from the first dose of lumasiran through January 12, 2024, are reported. AEs were classified using the Medical Dictionary for Regulatory Activities version 25.0 or later.

## Results

### Patients

Of 39 patients enrolled in the pivotal study, 24 of 26 (lumasiran/lumasiran group) and 13 of 13 (placebo/lumasiran group) completed treatment in the open-label extension (Supplemental Figure 1). BL characteristics were generally well balanced between groups (Supplemental Table 1).

### Efficacy

**Figure 1:**
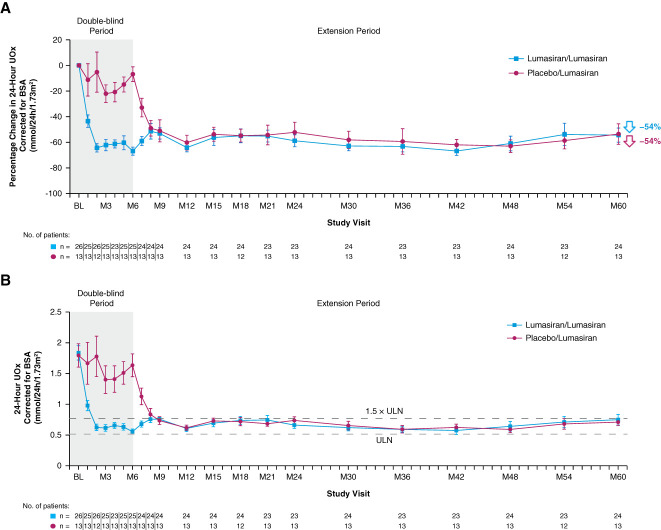
**Change in 24-hour UOx over time.** (A) Mean (SEM) percentage change in 24-hour UOx corrected for BSA. Values at Month 60 are indicated by arrows. (B) Mean (SEM) absolute values of 24-hour UOx corrected for BSA. Top dashed line represents 1.5 times ULN of 0.771 mmol/24 h per 1.73 m^2^ (1 mmol/24 h per 1.73 m^2^=90 mg/24 h per 1.73 m^2^) for 24-hour UOx excretion. Bottom dashed line represents the ULN of 0.514 mmol/24 h per 1.73 m^2^. BL is the median of all valid 24-hour urine assessments collected before the first dose date/time of study drug (lumasiran or placebo) without any non–protocol-related sample issues. BL, baseline; BSA, body surface area; M, month; ULN, upper limit of normal; UOx, urinary oxalate.

Sustained reductions in 24-hour UOx with long-term lumasiran treatment were observed in both treatment groups. At the end of the study (Month 60), the mean (SEM) 24-hour UOx percentage reductions were 54% (6%) in the lumasiran/lumasiran group and 54% (8%) in the placebo/lumasiran group relative to study BL (Figure 1A). Mean (SEM) 24-hour UOx (mmol/24 h per 1.73 m^2^) values decreased from 1.84 (0.12) at study BL to 0.75 (0.09) at Month 60 in the lumasiran/lumasiran group and 1.79 (0.19) at study BL to 0.71 (0.05) at Month 60 in the placebo/lumasiran group (Figure 1B). The mean 24-hour UOx values generally remained between the upper limit of normal (ULN; 0.514 mmol/24 h per 1.73 m^2^) and 1.5 times ULN (0.771 mmol/24 h per 1.73 m^2^) at all time points after approximately 2–3 months of lumasiran treatment. Similar reductions were observed in the spot UOx:Cr (Supplemental Figure 2).

**Figure 2:**
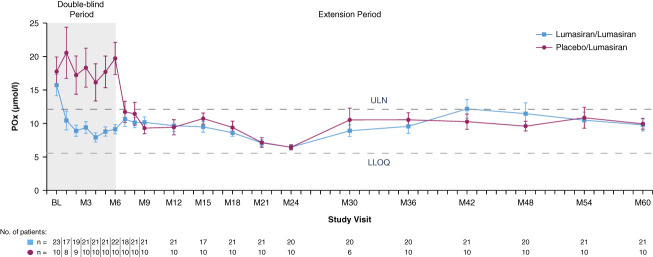
**Mean (SEM) POx concentration over time.** BL is defined as the mean of all POx measurements before the first dose date/time of study drug (lumasiran or placebo) in the 6-month DB period. Top dashed line represents the ULN of 12.11 *μ*mol/L for POx. Bottom dashed line represents the LLOQ of the POx assay at 5.55 *μ*mol/L; values below the LLOQ were assigned a value of 5.55 *μ*mol/L. DB, double-blind; LLOQ, lower limit of quantitation; POx, plasma oxalate.

The mean (SEM) POx concentrations (*µ*mol/L) decreased from 15.7 (1.6) at study BL to 9.8 (0.9) at Month 60 in the lumasiran/lumasiran group and from 17.8 (2.2) at study BL to 10.0 (0.8) at Month 60 in the placebo/lumasiran group (Figure 2). In both treatment groups, the mean POx concentrations were within normal limits (ULN, 12.11 *µ*mol/L) throughout the extension period, except for a mean value of 12.18 *µ*mol/L at Month 42 in the lumasiran/lumasiran group. The mean POx concentrations decreased from BL to Month 60 by 35% in the lumasiran/lumasiran group and 38% in the placebo/lumasiran group.

Plasma glycolate increased during the first 6 months of lumasiran treatment, then plateaued, and remained stable (Supplemental Figure 3). The mean (SEM) absolute changes in plasma glycolate concentration at Month 60 relative to study BL were 79.7 (10.7) *µ*mol/L in the lumasiran/lumasiran group and 97.0 (23.4) *µ*mol/L in the placebo/lumasiran group.

**Figure 3:**
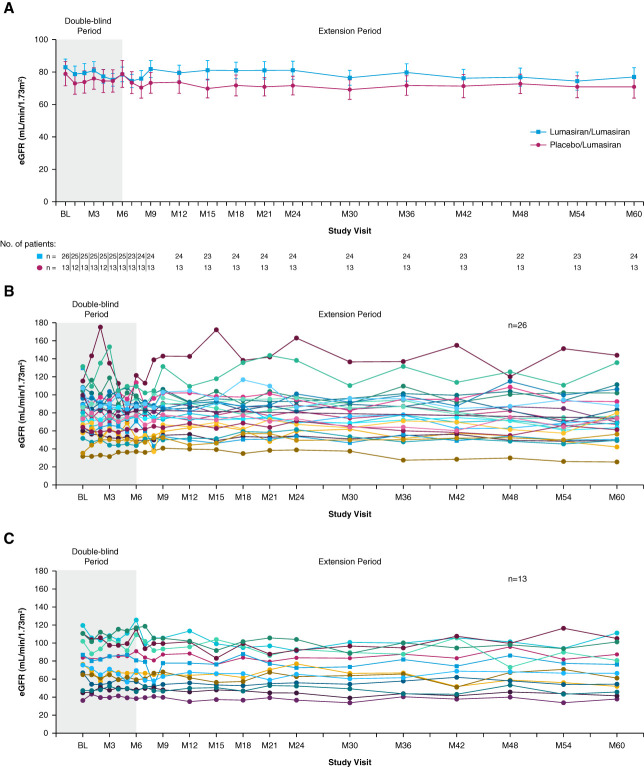
**eGFR over time.** (A) Mean (SEM) eGFR in the overall population, and spaghetti plots of actual values of eGFR in (B) the lumasiran/lumasiran group and (C) the placebo/lumasiran group. In the spaghetti plots, each line represents an individual patient. BL is the last assessment before the first dose of study drug (lumasiran or placebo) in the 6-month DB period.

The mean (SEM) absolute change in eGFR from study BL to Month 60 was −3.40 (2.49) ml/min per 1.73 m^2^ in the lumasiran/lumasiran group and −8.11 (2.25) ml/min per 1.73 m^2^ in the placebo/lumasiran group (Figure 3A). The regression-estimated annual rate of eGFR change over 60 months (slope [SEM]) for all patients in the study during lumasiran treatment (*N*=39) was −0.6 (0.7) ml/min per 1.73 m^2^/year. Plots of eGFR values in individual patients demonstrated little change over time, even among patients with lower eGFR values at BL (Figure 3, B and C).

**Figure 4:**
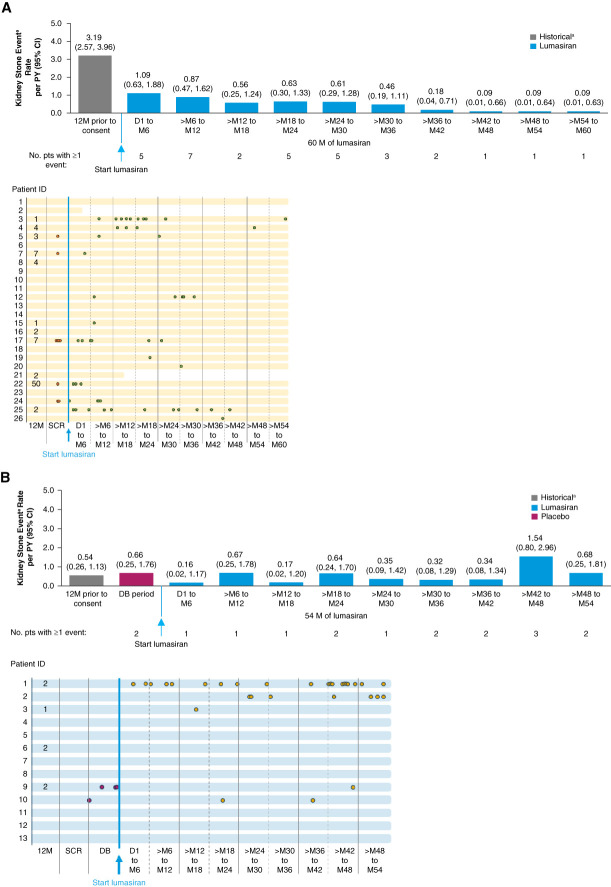
**Kidney stone events.** KSE rates in (A) the lumasiran/lumasiran group, overall (top) and in individual patients (bottom), and (B) the placebo/lumasiran group, overall (top) and in individual patients (bottom). A KSE is defined as an event that includes at least one of the following: a visit to a health care provider because of a kidney stone, medication for kidney colic, stone passage, or macroscopic hematuria due to a kidney stone. In the swim plots, each line represents one patient and each data point indicates one KSE (data points may overlap if events were close in time). The timing for historical events (before 12 months) was not documented. ^a^Patient-reported history of KSEs. CI, confidence interval; KSE, kidney stone event; PY, patient year.

KSE rates during the study were comparable to rates reported previously^26^: 0.47 (95% confidence interval, 0.36 to 0.62) per PY in the lumasiran/lumasiran group and 0.54 (0.37 to 0.78) per PY in the placebo/lumasiran group (Figure 4). Twenty-one (54%) patients had no KSEs, 7 (18%) patients had one KSE, and 11 (28%) patients had >1 KSE. The overall KSE rate in the placebo/lumasiran group was largely driven by one patient with a heavy stone burden and events that persisted through the extension period despite ongoing lumasiran treatment (Patient 1 in Figure 4B). KSEs in other patients in the placebo/lumasiran group were sparse.

**Figure 5:**
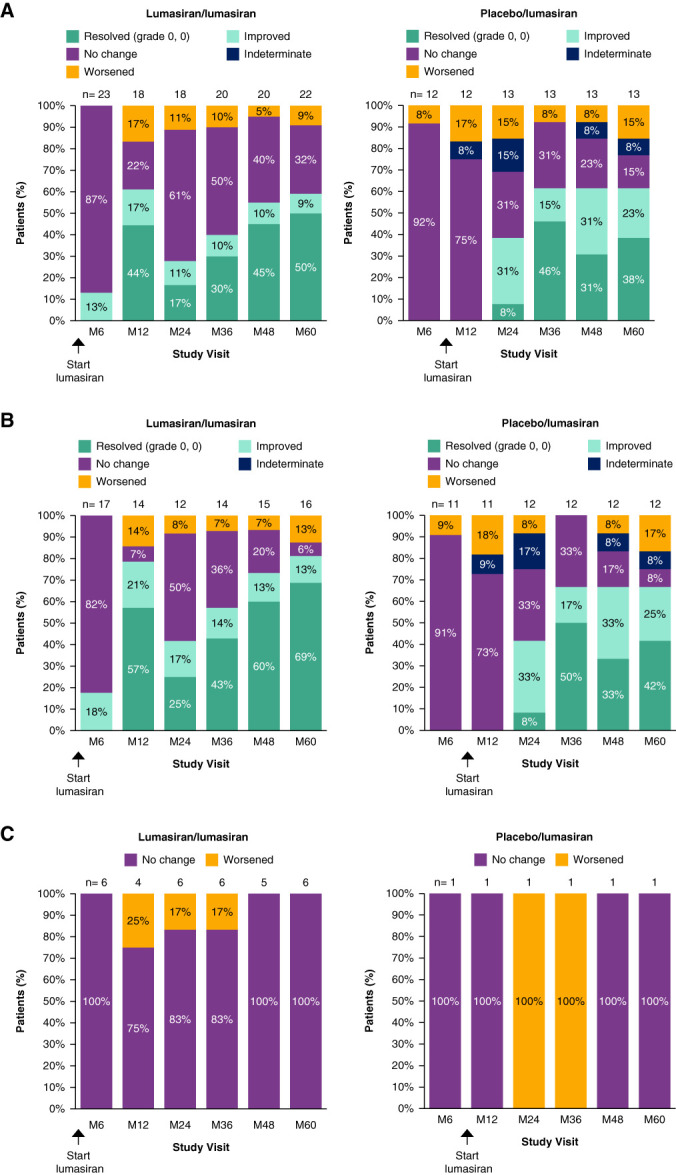
**Change in medullary nephrocalcinosis.** (A) All available patients, (B) patients with nephrocalcinosis at BL, and (C) patients without nephrocalcinosis at BL. Improved indicates a lower grade than BL (defined as the last assessment before the first dose of study drug [lumasiran or placebo]) in the 6-month DB period. No change indicates the same grade as BL. Indeterminate indicates one side improved and the other side worsened. Worsened indicates a higher grade than BL.

A total of 37 patients had kidney ultrasounds at BL, including 8 (22%) with bilateral grade 0 medullary nephrocalcinosis (*i.e*., none detected), 7 (19%) with bilateral grade 1 medullary nephrocalcinosis, 4 (11%) with bilateral grade 2, and 8 (22%) with bilateral grade 3 (severe). Medullary nephrocalcinosis grade (left/right or right/left) was 1/0 or 1/0 in 5 (14%) patients, 1/2 or 2/1 in 2 (5%) patients, 2/0 in 2 (5%) patients, and 2/3 in 1 (3%) patient. In patients with and without nephrocalcinosis at BL, nephrocalcinosis grade generally improved or remained stable (no change) at Month 60 relative to study BL (Figure 5). Among patients who had medullary nephrocalcinosis at BL and an assessment at end of study (*N*=28), medullary nephrocalcinosis grade improved in 21 (75%) patients and improved to complete resolution (grade 0, 0) in 16 (57%) patients. The percentage of patients with grade 0 nephrocalcinosis generally increased over time relative to the start of lumasiran treatment (Supplemental Figure 4).

Both treatment groups demonstrated maintenance in EQ-5D VAS scores (pooled results for EQ-5D-5L and EQ-5D-Y) during the extension period, with mean (SD) changes from BL at Month 60 of 1.8 (15.1) in the lumasiran/lumasiran group and −4.3 (24.9) in the placebo/lumasiran group (Supplemental Figure 5A). Scores for both adults and pediatric patients on the EQ-5D VAS (EQ-5D-5L and EQ-5D-Y) and EQ-5D-5L index all remained stable (Supplemental Figure 5, B–D). In addition, maintenance of HRQOL, as measured by the SF-12 physical and mental components of the KDQOL, was observed in both treatment groups during the extension period (Supplemental Figure 6, A–B). At Month 60, the lumasiran/lumasiran group demonstrated a mean (SD) change from study BL in KDQOL scores assessing kidney function through symptoms/problems (17.50 [31.98]), the effects of kidney disease (6.25 [16.24]), and the burden of kidney disease (12.50 [34.61]; Supplemental Figure 6, C–E). The lumasiran/lumasiran group also demonstrated a PedsQL total score mean (SD) change from study BL at Month 60 of 9.39 (18.60; Supplementary Figure 7).

All 39 patients enrolled in the study provided a BL sample for ADA testing, and 38 patients also provided at least one postdose sample. No patients tested positive for ADAs at BL. Two of 39 (5%) patients had a postdose sample that tested positive for ADAs: one patient (lumasiran/lumasiran) had a low titer (50) positive result at a single time point and the other patient (placebo/lumasiran) had low titers (100–50) over 18 months (Month 18 to Month 36), which then returned to negative through end of study. Both patients maintained their reductions in 24-hour UOx at the time points coinciding with titer positivity.

Overall, 31 (79%) of the 39 patients were under health care-provider instructions to hyperhydrate at BL. Most of those patients (28 of 31; 90%) continued to hyperhydrate for the duration of the study, and the rest (3 of 31; 10%) decreased hyperhydration during the study. No patients started hyperhydration if they were not hyperhydrating at BL.

### Safety

Overall, 37 (95%) patients had AEs (Table 1). The most common AEs by Medical Dictionary for Regulatory Activities preferred term, reported by ≥15% of patients during lumasiran treatment, were injection site reactions (36%), abdominal pain (23%), COVID-19 (21%), headache (18%), and nasopharyngitis (15%). Six (15%) patients had serious AEs; most resolved without sequelae and none led to discontinuation of study drug or withdrawal from the study. Most AEs were mild in severity (16 [41%] patients) or moderate in severity (17 [44%] patients). Four (10%) patients had severe AEs. One (3%) patient had an AE leading to discontinuation of study drug, which was not considered related to study drug. There were no deaths.

**Table 1:** Safety profile of lumasiran

Event, *n* (%)	Lumasiran/Lumasiran (*N*=26)	Placebo/Lumasiran (*N*=13)	All Lumasiran^a^ (*N*=39)
Any AE	25 (96)	12 (92)	37 (95)
AE related to study drug	13 (50)	6 (46)	19 (49)
Serious AE^b^	5 (19)	1 (8)	6 (15)
Severe AE^c^	4 (15)	0	4 (10)
AE leading to discontinuation of study treatment^d^	1 (4)	0	1 (3)
**AEs occurring in ≥15% of patients (during lumasiran treatment)**			
Injection site reactions^e^	9 (35)	5 (38)	14 (36)
Abdominal pain	8 (31)	1 (8)	9 (23)
COVID-19	4 (15)	4 (31)	8 (21)
Headache	5 (19)	2 (15)	7 (18)
Nasopharyngitis	4 (15)	2 (15)	6 (15)
Death	0	0	0

AE, adverse event; COVID-19, coronavirus disease 2019.

a Includes all patients who received any lumasiran during the study.

b Abdominal pain (*n*=2), dysuria (*n*=1), follicular lymphoma (*n*=1), postprocedural complication (*n*=1), postprocedural infection (*n*=1), kidney impairment (*n*=1), urinary tract infection (*n*=1), and urosepsis (*n*=1), all considered not related to lumasiran by the investigator.

c Acute pyelonephritis (*n*=1), follicular lymphoma (*n*=1), postprocedural complication (*n*=1), postprocedural infection (*n*=1), urinary tract infection (*n*=1), and urosepsis (*n*=1), all considered not related to lumasiran by the investigator.

d Fatigue and disturbance in attention, considered not related to lumasiran by the investigator, which began during the double-blind period.

e All were transient, considered mild in severity, and resolved without sequelae.

## Discussion

In this final, 60-month analysis of ILLUMINATE-A, patients with PH1 (aged ≥6 years) on long-term lumasiran treatment demonstrated substantial and sustained reductions in UOx excretion and POx concentration, encouraging clinical outcomes data, and an acceptable safety profile.

A sustained, clinically meaningful percentage change in 24-hour UOx corrected for body surface area was observed during the extension period in both treatment groups. Changes in 24-hour UOx excretion and percentage changes in spot 24-hour UOx:Cr and POx supported these findings. Levels of plasma glycolate initially increased and then plateaued in both treatment groups, as expected based on the mechanism of action of lumasiran.^9^ Hyperhydration is a supportive therapy that is typically initiated in patients with elevated UOx. Two case studies suggest that the need for hyperhydration may decline with decreasing UOx excretion secondary to RNAi treatment, but this should be interpreted with caution in the absence of stronger evidence.^27^ In this study, most patients who were hyperhydrating at BL continued to do so through the extension period, and no patients began hyperhydrating if they were not at BL.

Patients with PH1 are expected to gradually accumulate systemic oxalate deposits in the kidneys, retina, bones, joints, heart, blood vessels, skin, and central nervous system.^1^ Oxalate deposits in the kidneys affect kidney function and potentially lead to kidney stones and medullary nephrocalcinosis.^1^ In this study, there was no notable change in eGFR during the study in the lumasiran/lumasiran group. In the smaller placebo/lumasiran group, there was a small decrease in eGFR within the first year, after which eGFR was stable. KSEs were variable; however, the overall KSE rate declined, and most (54%) patients experienced no KSEs during lumasiran treatment. Patients also exhibited improvements in medullary nephrocalcinosis.

There were improvements in components of questionnaires such as the PedsQL and KDQOL related to symptoms and burden of kidney disease during the extension period, although the study was not designed to measure statistically significant changes in HRQOL. There is no available information on the minimal clinically important differences for these instruments in PH1. The minimally important difference (MID) for the EQ-5D VAS in pediatric patients (not specific to PH1) is dependent on BL VAS score; for BL scores in the range of those observed in this study (82.0–84.0), the estimated MID is 2.8–3.1.^28^ The mean (SD) changes from BL at Month 60 in EQ-5D VAS scores (1.8 [15.1] and −4.3 [24.9] in the lumasiran/lumasiran group and placebo/lumasiran group, respectively) did not meet this MID criterion. However, BL scores in this study are comparable with population norms,^29^ so improvement with lumasiran treatment is not to be expected, but the lack of worsening over the course of this 60-month study is encouraging. It is possible that the reduced rate of KSEs, which are considered a major determinant of PH1 disease burden, contributed to the observation that HRQOL was relatively stable or mildly improved during the study; another contributor may have been avoidance of initiation of hyperhydration among patients who were not hyperhydrating at BL, as hyperhydration has a negative effect on quality of life.^3,6,30^ The literature on HRQOL in PH1 is small but generally shows poor and/or worsening HRQOL in this patient population.^3,4^

Across both treatment groups, eGFR over the 60-month study period was virtually flat (mean [SEM] change, −0.6 [0.7] ml/min per 1.73 m^2^) in a population that would be expected to show eGFR decline. Historically, annual declines in eGFR of −2.3, −5.3, and −14.7 ml/min per 1.73 m^2^ with BL CKD stages 2, 3a, and 3b, respectively, have been observed in patients with PH1 who would have been eligible to participate in this study.^31^

Two patients who tested positive for ADAs after initiating lumasiran maintained their reductions in 24-hour UOx at time points coinciding with titer positivity, indicating that ADAs did not affect lumasiran's efficacy on this outcome.

Lumasiran safety data were consistent with previous observations. Most AEs were mild or moderate in severity. Six patients had at least one serious AE during the study; most resolved without sequelae, and none were assessed to be related to the study drug. No deaths were reported. The most commonly reported related AEs were injection site reactions, as reported previously in the phase 2 open-label extension study of lumasiran in PH1 (24-month analysis).^32^ No new safety signals were observed.

This study has a number of strengths. First, the demographic and BL disease characteristics of the study population were representative of the general PH1 population. Second, the duration of lumasiran treatment was the longest to date for an RNAi treatment in PH1 (54–60 months), allowing for observation of long-term maintenance of lumasiran treatment effects. Third, patient retention was high, with 37 of 39 patients enrolled in the pivotal study completing treatment in the extension phase. Fourth, multiple outcome measures were used to examine symptoms and outcomes of PH1.

This study also has limitations. First, as is typical for an extension study, there was no placebo group or blinding in the extension period. Second, patients younger than 6 years and with more severe disease (eGFR<30 ml/min per 1.73 m^2^) were excluded. However, young patients are included in ILLUMINATE-B and ILLUMINATE-C, although patients in ILLUMINATE-B were required to have relatively more preserved kidney function.^10,33^ The lumasiran clinical development program captures the full spectrum of patient ages and PH1 disease severity. In addition, an observational study of lumasiran in PH1 (BONAPH1DE) is underway, complementing randomized controlled trials.^34^ Third, the KSE rate during the 12 months before study entry was reported retrospectively by patients and is subject to error and recall bias. Finally, this study was not designed to evaluate the statistical significance of changes in HRQOL.

In conclusion, lumasiran treatment for up to 60 months in ILLUMINATE-A was associated with sustained reductions in UOx excretion and POx concentration, encouraging clinical outcomes including stable eGFR, reduced KSEs, improved medullary nephrocalcinosis, indications of improvement in HRQOL, and acceptable safety.

## Supplementary Material

## Data Availability

Access to anonymized individual participant data that support these results is made available 12 months after study completion and not <12 months after the product and indication have been approved in the United States and/or the EU. Data will be provided contingent on the approval of a research proposal and the execution of a data sharing agreement. Requests for access to data can be submitted through the website www.vivli.org.

## Declarative Statements

This study includes clinical experimentation and received Institutional Review Board or Ethics Committee approval. All patients provided written informed consent. This study includes clinical experimentation and complies with the Declaration of Helsinki.

## Supplemental Material

Supplemental Table 1. BL characteristics.

Supplemental Figure 1. Patient disposition.

Supplemental Figure 2. Change in spot UOx:Cr over time. (A) Mean (SEM) percentage change in spot UOx:Cr. (B) Mean (SEM) absolute values of spot UOx:Cr.

Supplemental Figure 3. Mean (SEM) plasma glycolate over time.

Supplemental Figure 4. Medullary nephrocalcinosis grade of left and right kidneys in (A) the lumasiran/lumasiran group and (B) the placebo/lumasiran group.

Supplemental Figure 5. Mean (SD) scores on (A) EQ VAS (pooled), (B) EQ VAS (adults), (C) EQ-5D-5L index, and (D) EQ VAS (pediatric).

Supplemental Figure 6. Mean (SD) scores on KDQOL subscales of (A) SF-12 physical component summary, (B) SF-12 mental component summary, (C) Symptoms/Problems, (D) Effects of Kidney Disease, and (E) Burden of Kidney Disease.

Supplemental Figure 7. Mean (SD) PedsQL and PedsQL ESKD summary scores, including (A) PedsQL total score, (B) PedsQL physical health summary score, (C) PedsQL psychosocial health summary score, (D) PedsQL ESKD total score (Patient), and (E) PedsQL ESKD total score (Parent).
