# Supplementary material for: Final Results of the ILLUMINATE-A Phase 3 Clinical Trial of Lumasiran for Primary Hyperoxaluria 1
Source: Clin J Am Soc Nephrol. 2025 Dec 4;21(3):377–88. doi: 10.2215/CJN.0000000916 (PMC12959742; doi:10.2215/CJN.0000000916)
Supplement: Supplementary file 1 [file cjasn-21-377-s001.pdf]

SUPPLEMENTAL MATERIAL

Final Results of the ILLUMINATE-A Phase 3 Clinical Trial of Lumasiran for Primary Hyperoxaluria 1

Yaacov Frishberg[1], Jeffrey M. Saland[2], John C. Lieske[3], Weiming Du[4], Martin Coenen[5], Julien Hogan[6], Anne-Laure Sellier-Leclerc[7], Jaap W. Groothoff[8], Cristin Kaspar[4], John M. Gansner[4], Sally-Anne Hulton[9] on behalf of the ILLUMINATE-A study investigators

[1]Division of Pediatric Nephrology, Shaare Zedek Medical Center, Jerusalem, Israel Faculty of Medicine, Hebrew University, Jerusalem, Israel; [2]Icahn School of Medicine at Mount Sinai, New York, NY, USA; [3]Division of Nephrology and Hypertension, Mayo Clinic, Rochester, MN, USA; [4]Alnylam Pharmaceuticals, Cambridge, MA, USA; [5]Institute of Clinical Chemistry and Clinical Pharmacology, University Hospital Bonn, Bonn, Germany; [6]Pediatric Nephrology Department, Hôpital Robert-Debré, APHP, Centre Référence Maladie Rares MARHEA, ERKnet, Paris, France; [7]Hôpital Femme Mère Enfant en Centre d'Investigation Clinique, Institut National de la Santé et de la Recherche Médicale (INSERM), Hospices Civils de Lyon, ERKnet, Bron, France; [8]Department of Pediatric Nephrology, Emma Children’s Hospital, Amsterdam UMC, University of Amsterdam, The Netherlands; [9]Department of Nephrology, Birmingham Women’s and Children's Hospital, Birmingham, UK

Corresponding Author:

Yaacov Frishberg

Division of Pediatric Nephrology, Shaare Zedek Medical Center, Jerusalem, Israel

Phone: 972-50-8685021

E-mail: yaacovf@ekmd.huji.ac.il

SUPPLEMENTAL MATERIAL

Contents

Supplemental Table 1. BL characteristics.

Supplemental Figure 1. Patient disposition.

Supplemental Figure 2. Change in spot UOx:Cr over time. (A) Mean (SEM) percentage change in spot UOx:Cr. (B) Mean (SEM) absolute values of spot UOx:Cr.

Supplemental Figure 3. Mean (SEM) plasma glycolate over time.

Supplemental Figure 4. Medullary nephrocalcinosis grade of left and right kidneys in (A) the lumasiran/lumasiran group and (B) the placebo/lumasiran group.

Supplemental Figure 5. Mean (SD) scores on (A) EQ VAS (pooled), (B) EQ VAS (adults), (C) EQ-5D-5L index, and (D) EQ VAS (pediatric).

Supplemental Figure 6. Mean (SD) scores on KDQOL subscales of (A) SF-12 PCS, (B) SF-12 MCS, (C) Symptoms/Problems, (D) Effects of Kidney Disease, and (E) Burden of Kidney Disease.

Supplemental Figure 7. Mean (SD) PedsQL and PedsQL ESKD summary scores, including (A) PedsQL total score, (B) PedsQL physical health summary score, (C) PedsQL psychosocial health summary score, (D) PedsQL ESKD total score (Patient), and (E) PedsQL ESKD total score (Parent).

Supplemental Table 1. Baseline characteristics.

Baseline Characteristic	Lumasiran/Lumasiran (n=26)	Placebo/Lumasiran (n=13)	All Lumasiran (n=39)
Age at informed consent, mean (range), years	18.7 (6–47)	17.0 (6–60)	18.1 (6–60)
Male, n (%)	18 (69)	8 (62)	26 (67)
Race, n (%)			
Asian	3 (12)	3 (23)	6 (15)
Other, or >1 race	2 (8)	1 (8)	3 (8)
White	21 (81)	9 (69)	30 (77)
Genotype[a], n (%)			
PR/*	11 (42)	6 (46)	17 (44)
M/M or M/N	6 (23)	4 (31)	10 (26)
N/N	9 (35)	3 (23)	12 (31)
Pyridoxine use[b], n (%)	13 (50)	9 (69)	22 (56)
24-hour UOx excretion corrected for BSA[c], mean (SD), mmol/24h/1.73m^2	1.8 (0.6)	1.8 (0.7)	1.8 (0.6)
POx, mean (SD), µmol/L[d]	14.8 (7.6)	16.5 (7.3)	15.0 (7.4)
eGFR, mean (SD), mL/min/1.73m^2	83.0 (25.5)	78.9 (26.8)	81.6 (25.7)
Patients reporting history of KSEs[e], n (%)			
Lifetime	23 (88)	10 (77)	33 (85)
12 months prior to consent	11 (42)	4 (31)	15 (38)

[a]PR was defined as NM_000030.3(AGXT):c.508G>A (p.Gly170Arg) or NM_000030.3(AGXT):c.454T>A (p.Phe152Ile). M and N were defined based on a publication by Mandrile et al.[1] The asterisk (*) denotes any genotype of PR, M, or N. M, missense; N, nonsense; PR, pyridoxine-responsive.

[b]Patients taking pyridoxine for the treatment of PH1 were required to have been on a stable regimen for at least 90 days before randomization, and willing to remain on this stable regimen for 12 months from first study drug administration. A total of 20 patients were taking pyridoxine medications at baseline and entered the extension period, including 11 in the lumasiran/lumasiran group and nine in the placebo/lumasiran group. Among them, two stopped taking pyridoxine during the extension period (one at Month 19 and one at Month 11) and did not resume prior to Month 36, and one had a ~two week gap in pyridoxine treatment during the

extension period (beginning at Day 187, or Month six). All others remained on pyridoxine treatment continuously through Month 60.
[c]ULN is 0.514 mmol/24h/1.73m^2 = 45 mg/24h/1.73m^2 (1 mmol/24h/1.73m^2 = 88 mg/24h/1.73m^2).
[d]ULN is 12.11 µmol/L.
[e]KSE is defined as an event that includes at least one of the following: a visit to a healthcare provider because of a kidney stone, medication for renal colic, stone passage, or macroscopic hematuria due to a kidney stone.
BSA, body surface area; eGFR, estimated glomerular filtration rate; KSE, kidney stone event; POx, plasma oxalate; SD, standard deviation; UOx, urinary oxalate.

Reference

1. Mandrile G, van Woerden CS, Berchialla P, et al. Data from a large European study indicate that the outcome of primary hyperoxaluria type 1 correlates with the AGXT mutation type. *Kidney Int.* 2014;86(6):1197-1204. 10.1038/ki.2014.222

Supplemental Figure 1. Patient disposition.

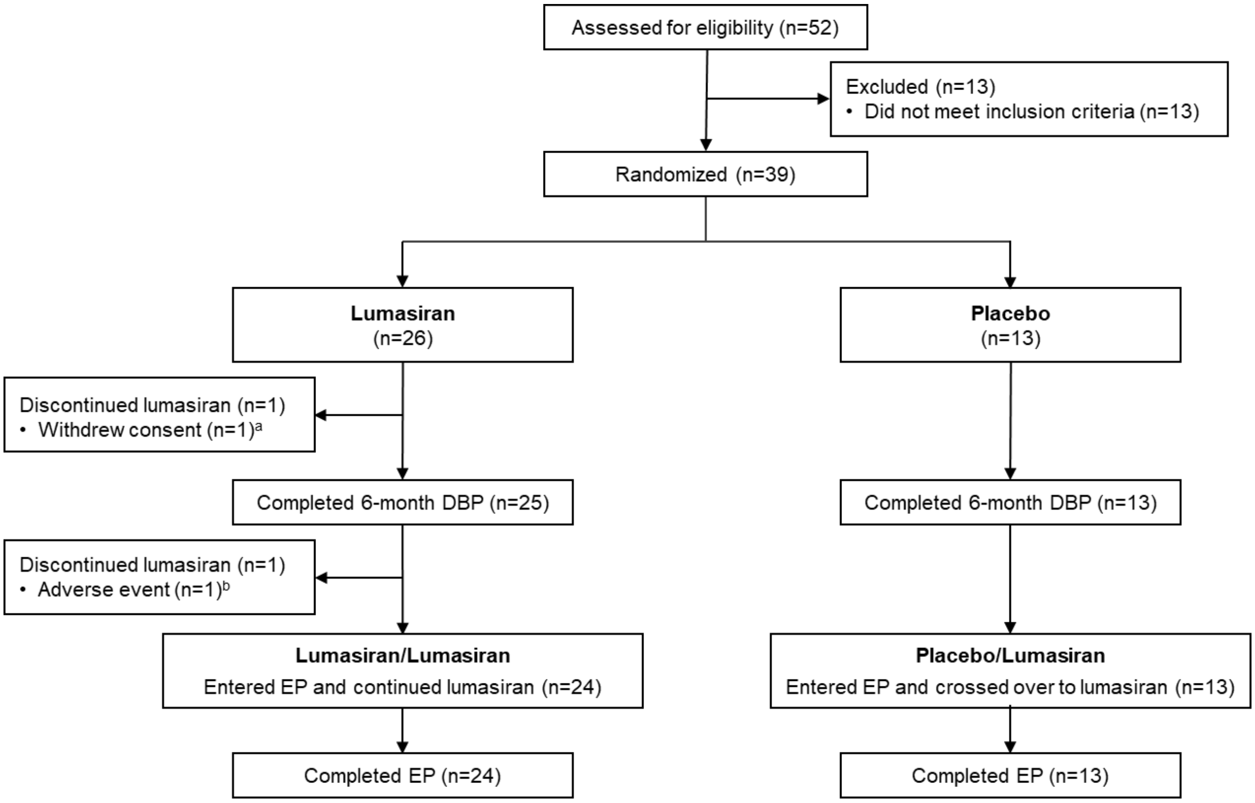

DBP, double-blind period; EP, extension period.
[a]Participation stopped by parent/guardian owing to the patient's inability to comply with protocol-specific testing; patient did not complete six-month DBP.
[b]Discontinued treatment for adverse events (unrelated to treatment) of fatigue and disturbance in attention; completed six-month DBP but did not enter EP.

Supplemental Figure 2. Change in spot UOx:Cr ratio over time. (A) Mean (SEM) percentage change in spot UOx:Cr. (B) Mean (SEM) absolute values of spot UOx:Cr.

A

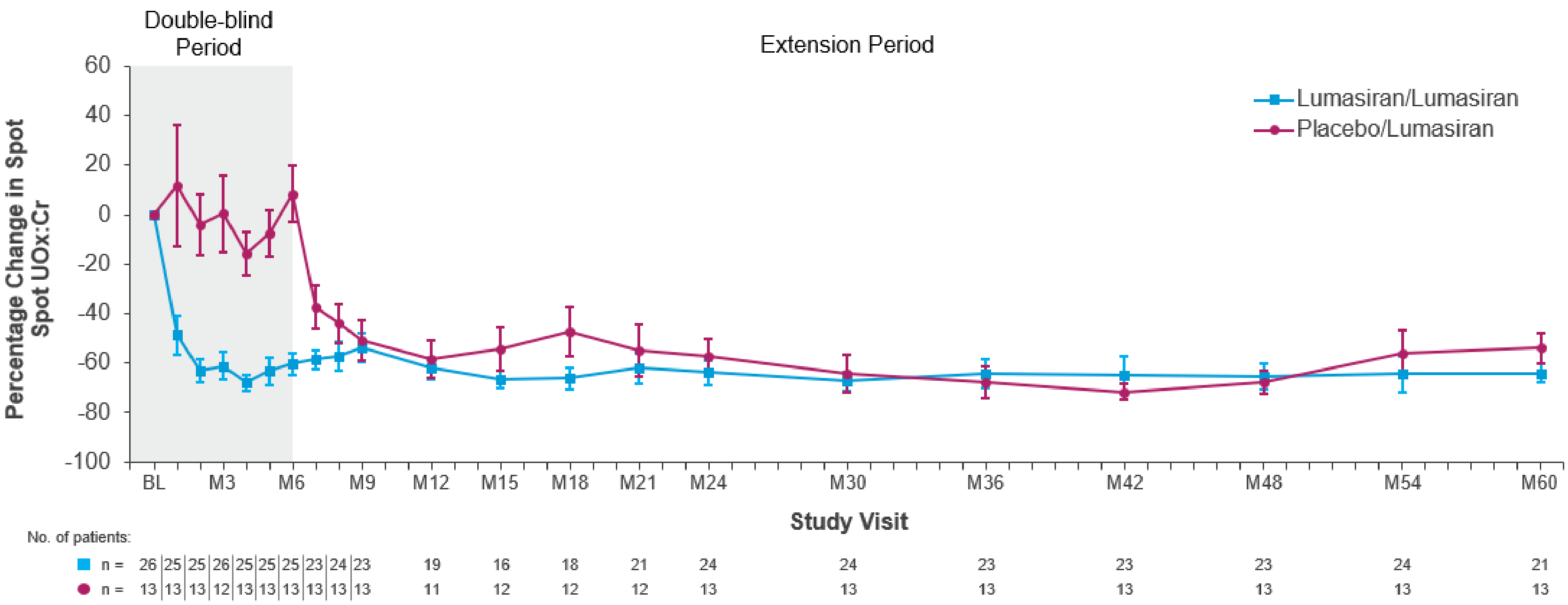

B

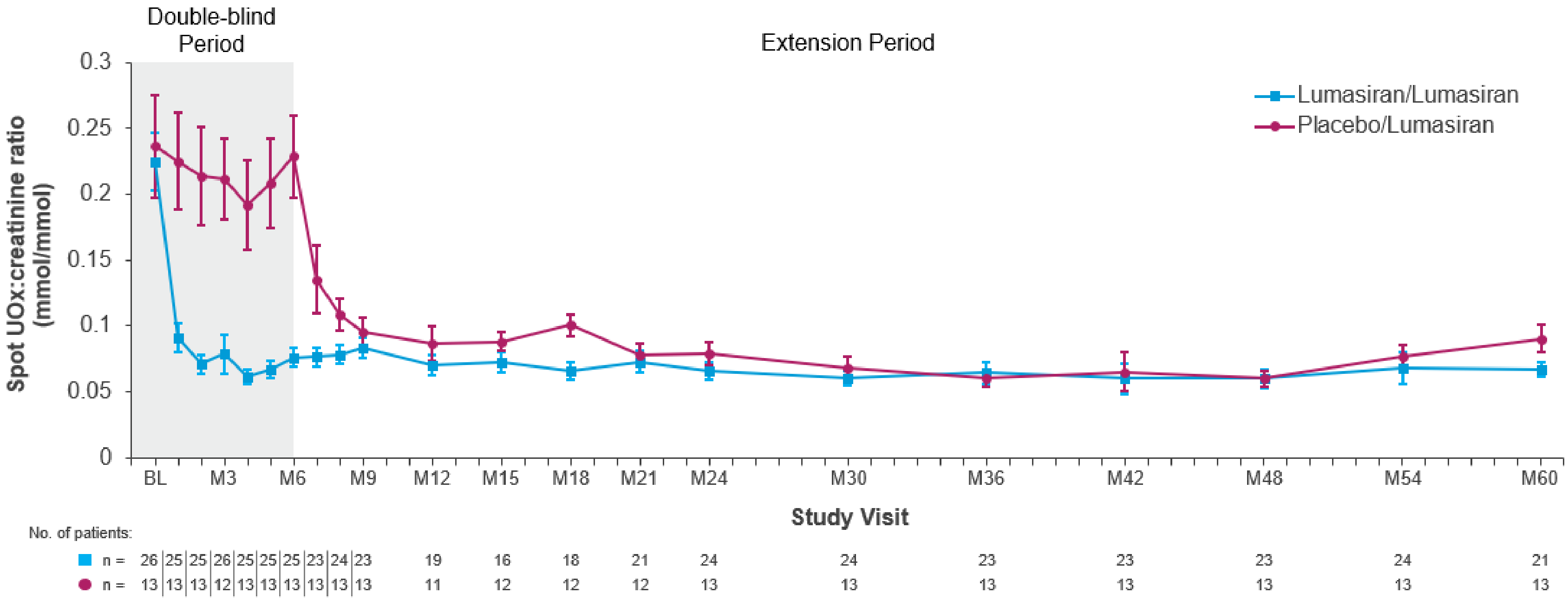

Baseline is defined as the mean of all measurements prior to the first dose date/time of study drug (lumasiran or placebo) in the six-month double-blind period.
BL, baseline; Cr: creatinine; M, month; SEM, standard error of mean; UOx, urinary oxalate.

Supplemental Figure 3. Mean (SEM) plasma glycolate over time.

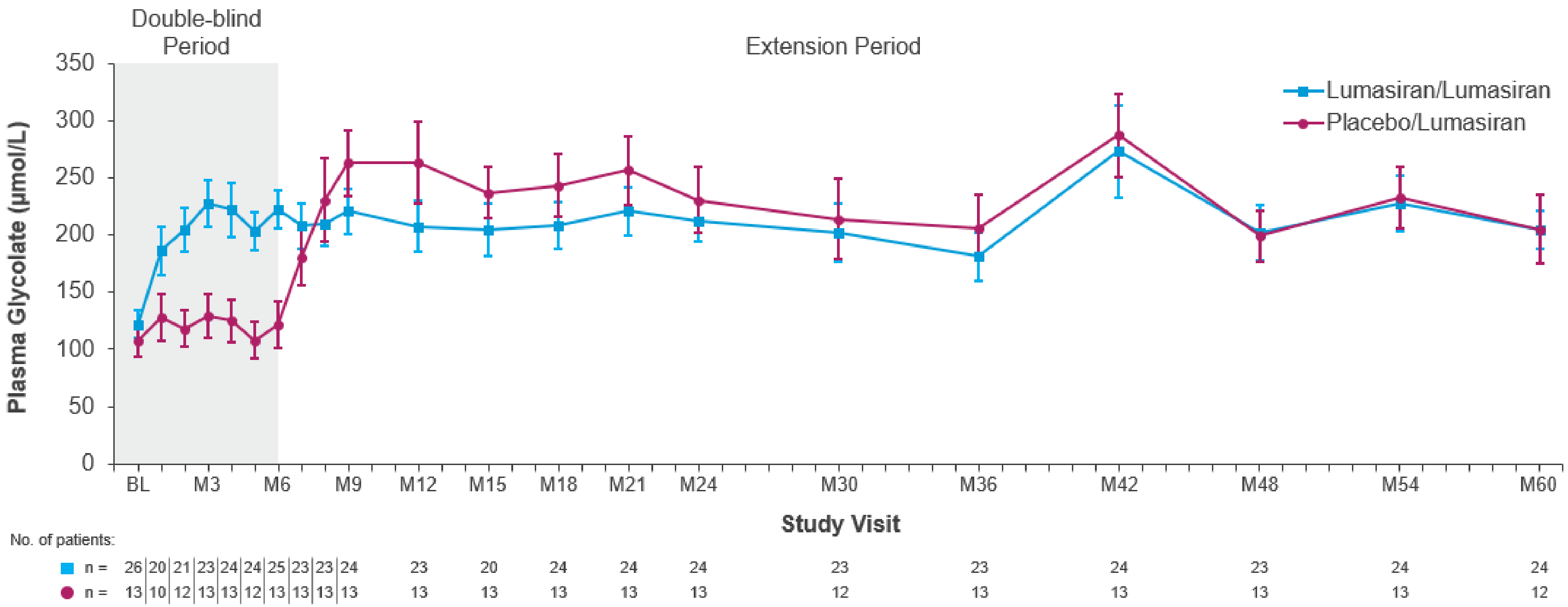

Baseline is defined as the mean of all measurements prior to the first dose date/time of study drug (lumasiran or placebo) in the six-month double-blind period.
BL, baseline; M, month; SEM, standard error of mean.

Supplemental Figure 4. Medullary nephrocalcinosis grade of left and right kidneys in (A) the lumasiran/lumasiran group and (B) the placebo/lumasiran group.

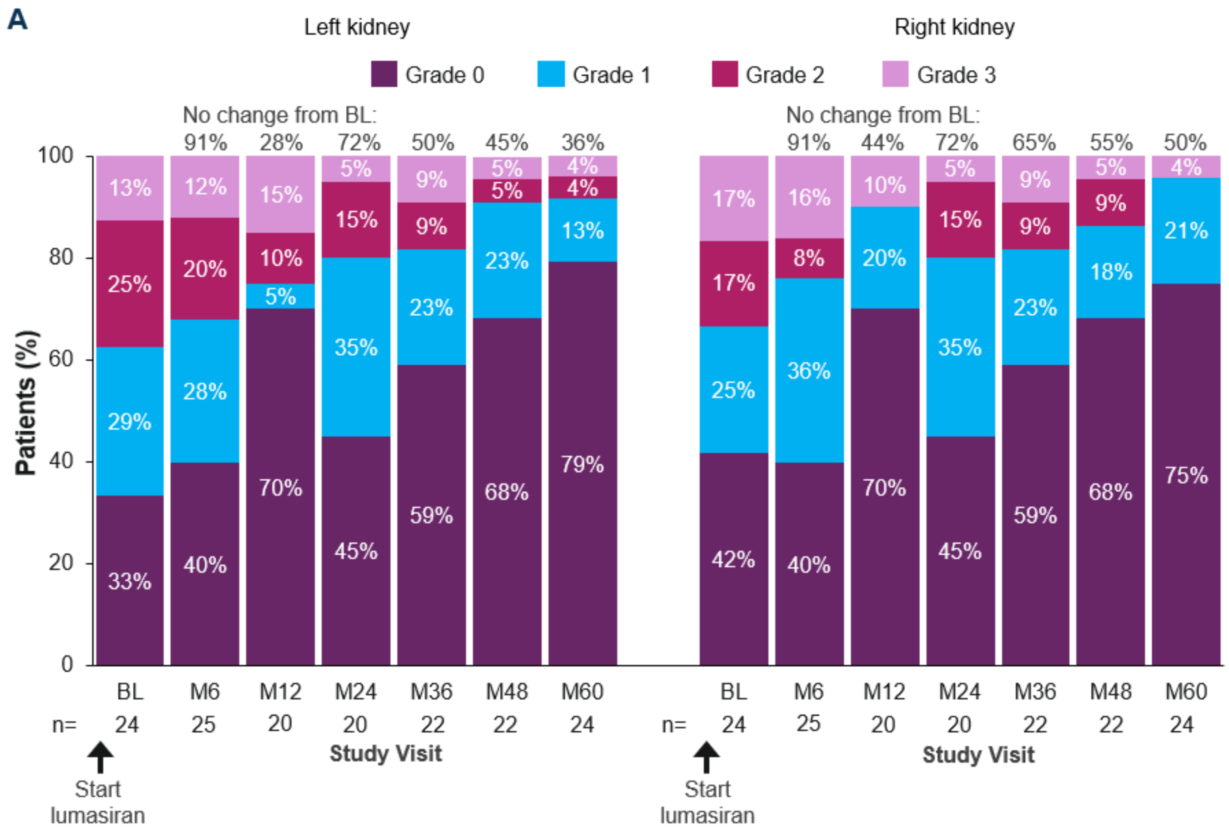

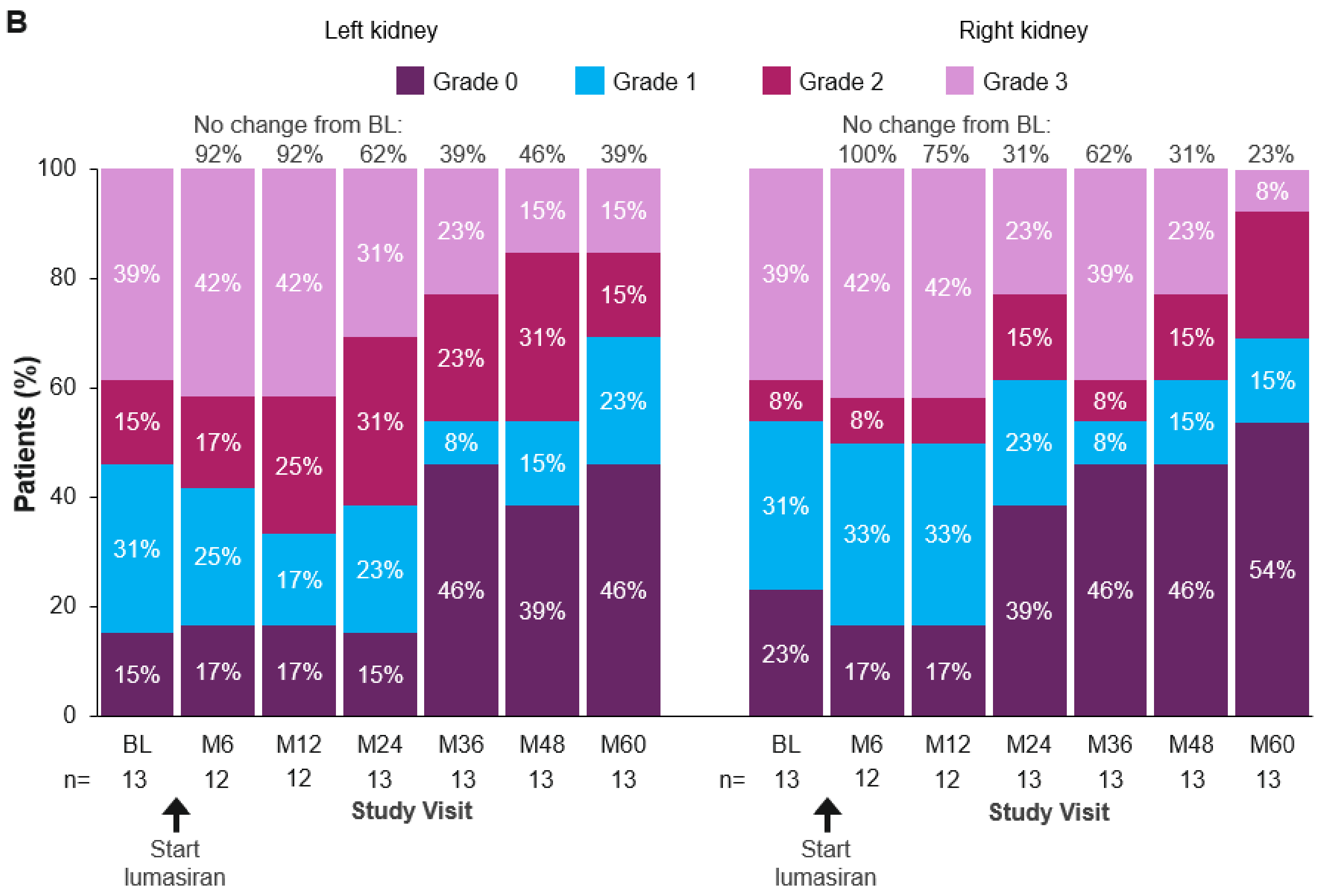

No change indicates the same grade as baseline (defined as the last assessment prior to the first dose of study drug [lumasiran or placebo] in the six-month double-blind period).

Supplemental Figure 5. Mean (SD) scores on (A) EQ VAS (pooled), (B) EQ VAS (adults), (C) EQ-5D-5L Index, and (D) EQ VAS (pediatric).

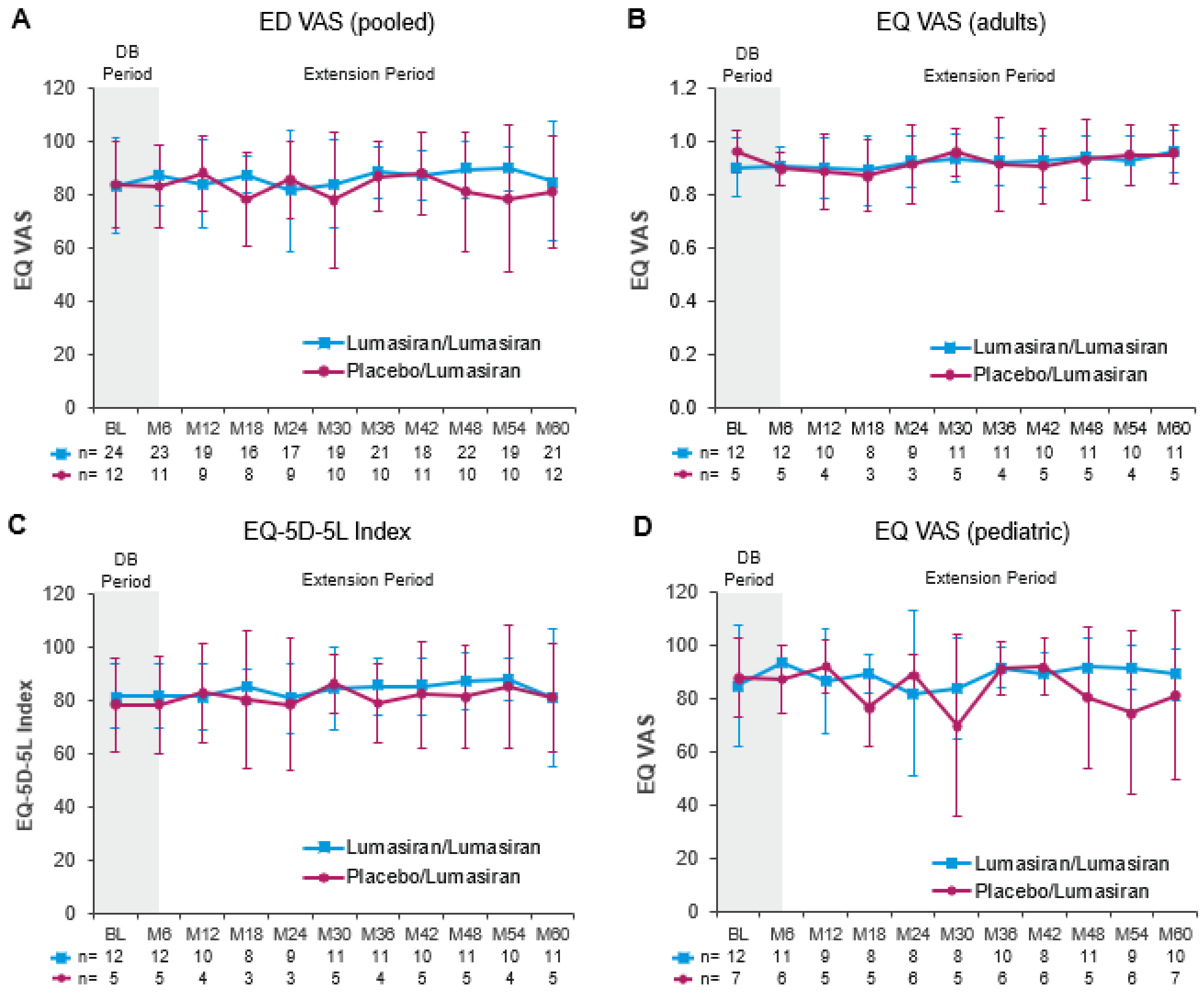

Baseline is the last assessment prior to the first dose of study drug (lumasiran or placebo) in the six-month double-blind period.
BL, baseline; EQ5D-VAS, Euro Quality of Life Health State Profile Questionnaire and Visual Analog Scale; M, month; SD, standard deviation.

Supplemental Figure 6. Mean (SD) scores on KDQOL[a] subscales of (A) SF-12 PCS, (B) SF-12 MCS, (C) Symptoms/Problems, (D) Effects of Kidney Disease, and (E) Burden of Kidney Disease.

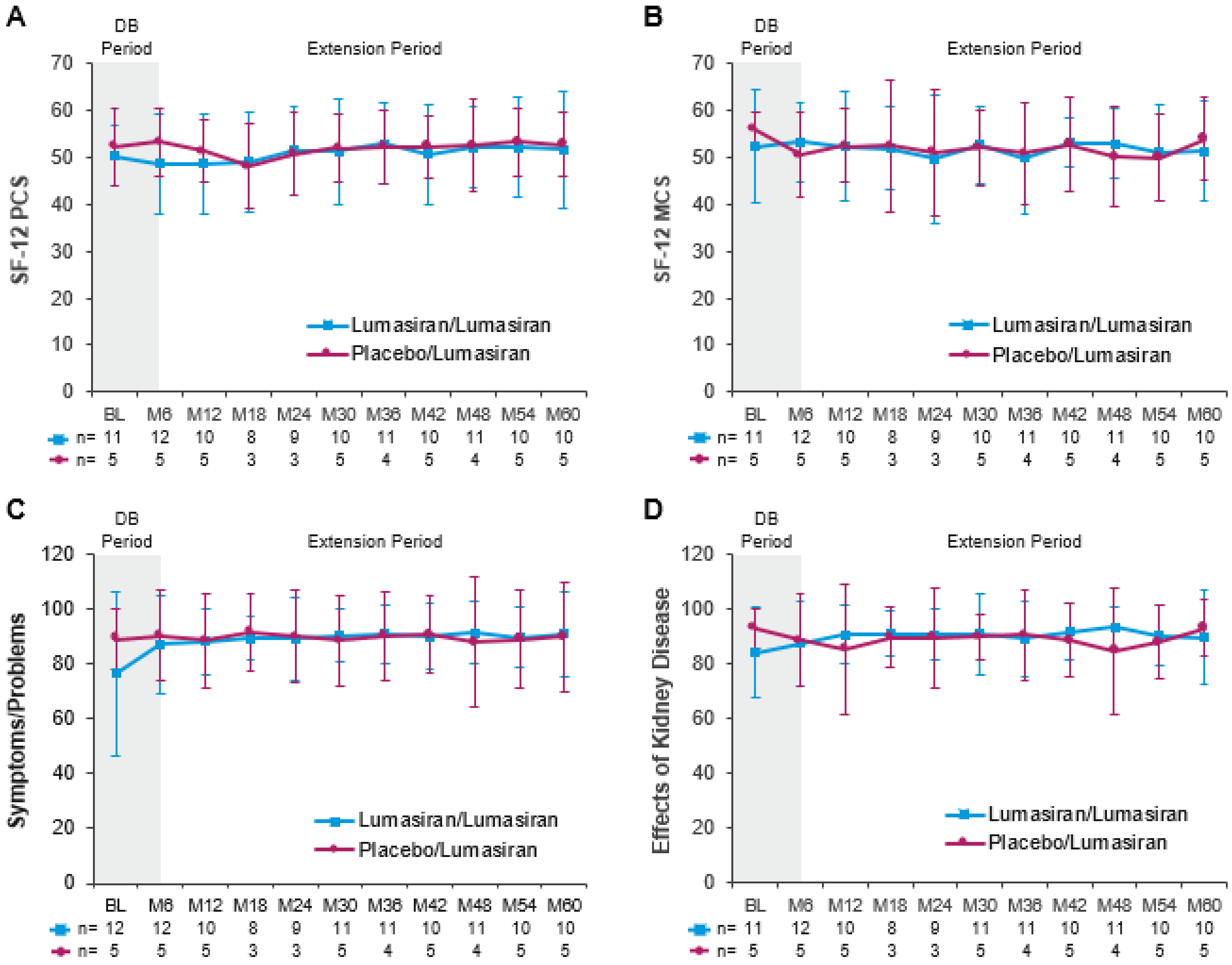

E

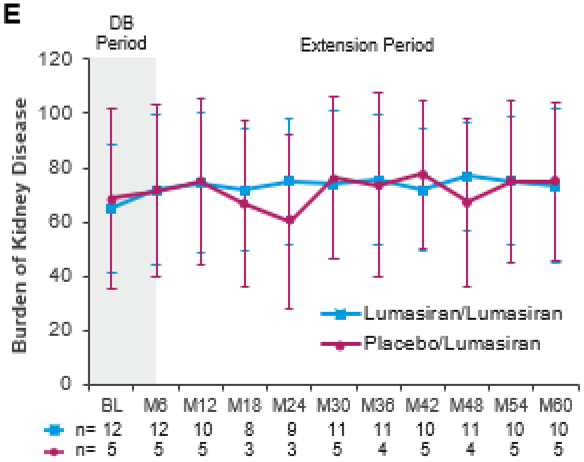

[a]Completed by patients aged ≥18 years.
Baseline is the last assessment prior to the first dose of study drug (lumasiran or placebo) in the six-month double-blind period.
BL, baseline; DB, double-blind; KDQOL, Kidney Disease Quality of Life Questionnaire; M, month; MCS, mental component summary; PCS, physical component summary; PedsQL; Pediatric Quality of Life Inventory; SD, standard deviation; SF-12, 12-item Short Form Survey.

Supplemental Figure 7. Mean (SD) PedsQL[a] and PedsQL ESKD summary scores, including (A) PedsQL total score, (B) PedsQL physical health summary score, (C) PedsQL psychosocial health summary score, (D) PedsQL ESRD total score (Patient)[a], and (E) PedsQL ESRD total score (Parent).

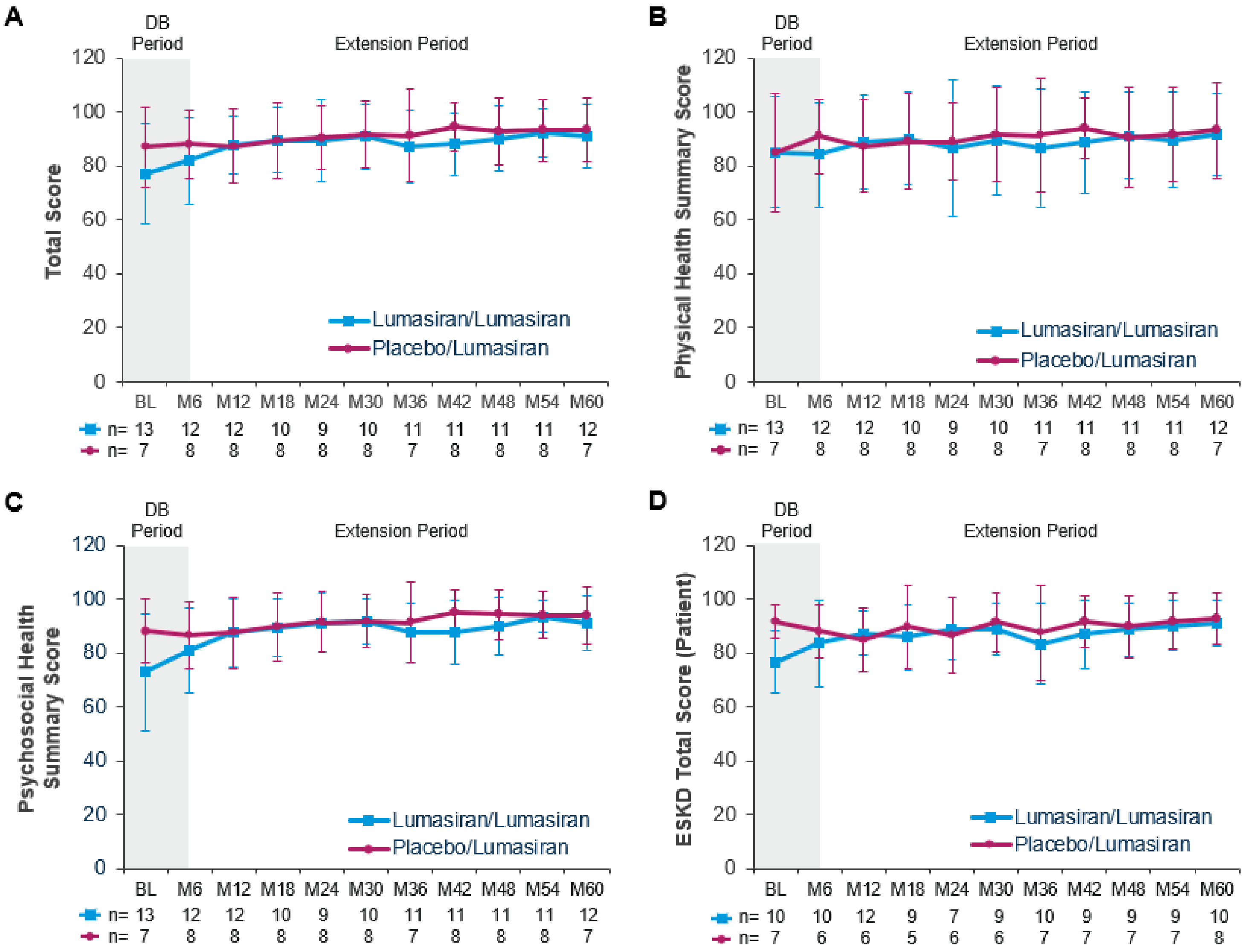

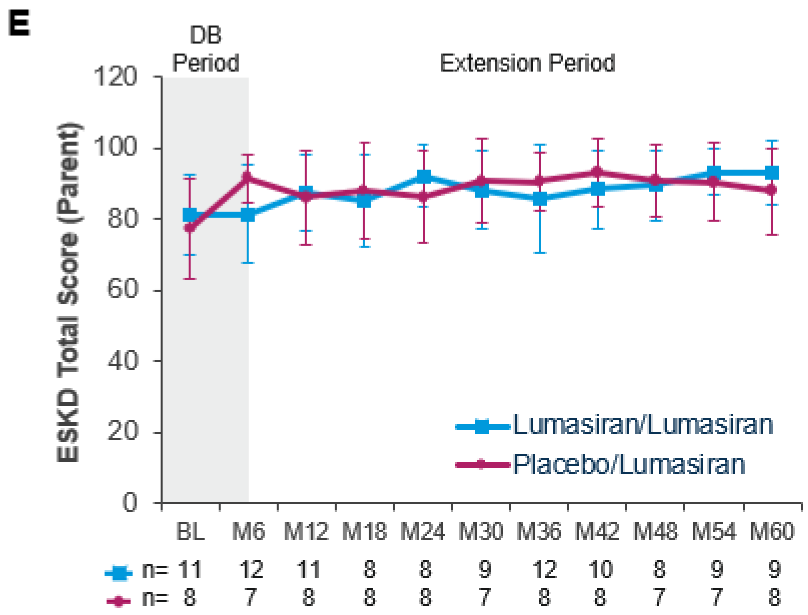

[a]Completed by patients aged <18 years old.
Baseline is the last assessment prior to the first dose of study drug (lumasiran or placebo) in the six-month double-blind period.
BL, baseline; DB, double-blind; ESRD, end stage renal disease; M, month; PedsQL, Pediatric Quality of Life Inventory; SD, standard deviation.
