# Supplementary material for: Final Results of the ILLUMINATE-A Phase 3 Clinical Trial of Lumasiran for Primary Hyperoxaluria 1
Source: Clin J Am Soc Nephrol. 2025 Dec 4;21(3):377–88. doi: 10.2215/CJN.0000000916 (PMC12959742; doi:10.2215/CJN.0000000916)
Supplement: Supplementary file 2 [file cjasn-21-377-s002.pdf]

ASN American Society of Nephrology JASN Journal of the American Society of Nephrology KIDNEY360 Accessing Our World From Every Angle CJASN Clinical Journal of the American Society of Nephrology

ASN Journal Disclosure Form

As per ASN journal policy, I have disclosed any financial relationships or commitments I have held in the past 36 months as included below. I have listed my Current Employer below to indicate there is a relationship requiring disclosure. If no relationship exists, my Current Employer is not listed.

M. Coenen reports the following:
Employer: University Hospital Bonn; and Research Funding: Günenthal.

I understand that the information above will be published within the journal article, if accepted, and that failure to comply and/or to accurately and completely report the potential financial conflicts of interest could lead to the following: 1) Prior to publication, article rejection, or 2) Post-publication, sanctions ranging from, but not limited to, issuing a correction, reporting the inaccurate information to the authors' institution, banning authors from submitting work to ASN journals for varying lengths of time, and/or retraction of the published work.

Name: Martin Coenen
Manuscript ID: CJASN-2025-000806
Manuscript Title: Sixty-Month Follow-Up of the ILLUMINATE-A Phase 3 Study of Lumasiran, the First Approved Therapeutic for Primary Hyperoxaluria 1
Date of Completion: August 4, 2025
Disclosure Updated Date: August 4, 2025

ASN American Society of Nephrology | JASN Journal of the American Society of Nephrology | KIDNEY360 Accessing Our World From Every Angle | CJASN Clinical Journal of the American Society of Nephrology

ASN Journal Disclosure Form

As per ASN journal policy, I have disclosed any financial relationships or commitments I have held in the past 36 months as included below. I have listed my Current Employer below to indicate there is a relationship requiring disclosure. If no relationship exists, my Current Employer is not listed.

W. Du reports the following:
Employer: Employee of and shareholder in Alnylam Pharmaceuticals.

I understand that the information above will be published within the journal article, if accepted, and that failure to comply and/or to accurately and completely report the potential financial conflicts of interest could lead to the following: 1) Prior to publication, article rejection, or 2) Post-publication, sanctions ranging from, but not limited to, issuing a correction, reporting the inaccurate information to the authors' institution, banning authors from submitting work to ASN journals for varying lengths of time, and/or retraction of the published work.

Name: Weiming Du
Manuscript ID: CJASN-2025-000806
Manuscript Title: Sixty-Month Follow-Up of the ILLUMINATE-A Phase 3 Study of Lumasiran, the First Approved Therapeutic for Primary Hyperoxaluria 1
Date of Completion: August 7, 2025
Disclosure Updated Date: August 7, 2025

ASN American Society of Nephrology JASN Journal of the American Society of Nephrology KIDNEY360 Accessing Our World From Every Angle CJASN Clinical Journal of the American Society of Nephrology

ASN Journal Disclosure Form

As per ASN journal policy, I have disclosed any financial relationships or commitments I have held in the past 36 months as included below. I have listed my Current Employer below to indicate there is a relationship requiring disclosure. If no relationship exists, my Current Employer is not listed.

Y. Frishberg reports the following:
Employer: Shaare Zedek Medical Center; Consultancy: Alnylam Pharmaceuticals; Arbor Biotechnologies; Honoraria: Alnylam Pharmaceuticals; and Advisory or Leadership Role: Alnylam Pharmaceuticals, member, Safety Review Committee;.

I understand that the information above will be published within the journal article, if accepted, and that failure to comply and/or to accurately and completely report the potential financial conflicts of interest could lead to the following: 1) Prior to publication, article rejection, or 2) Post-publication, sanctions ranging from, but not limited to, issuing a correction, reporting the inaccurate information to the authors' institution, banning authors from submitting work to ASN journals for varying lengths of time, and/or retraction of the published work.

Name: Yaacov Frishberg
Manuscript ID: CJASN-2025-000806
Manuscript Title: Sixty-month follow-up of the Illuminate A Phase 3 study of lumasiran, the first approved therapeutic for primary hyperoxaluria 1
Date of Completion: July 14, 2025
Disclosure Updated Date: July 14, 2025

ASN American Society of Nephrology
JASN Journal of the American Society of Nephrology
KIDNEY360 Accessing Our World From Every Angle
CJASN Clinical Journal of the American Society of Nephrology

ASN Journal Disclosure Form

As per ASN journal policy, I have disclosed any financial relationships or commitments I have held in the past 36 months as included below. I have listed my Current Employer below to indicate there is a relationship requiring disclosure. If no relationship exists, my Current Employer is not listed.

J. Gansner reports the following:
Employer: Alnylam Pharmaceuticals; and Ownership Interest: Alnylam Pharmaceuticals, Beam Therapeutics.

I understand that the information above will be published within the journal article, if accepted, and that failure to comply and/or to accurately and completely report the potential financial conflicts of interest could lead to the following: 1) Prior to publication, article rejection, or 2) Post-publication, sanctions ranging from, but not limited to, issuing a correction, reporting the inaccurate information to the authors' institution, banning authors from submitting work to ASN journals for varying lengths of time, and/or retraction of the published work.

Name: John M. Gansner
Manuscript ID: CJASN-2025-000806R1
Manuscript Title: Sixty-Month Follow-Up of the ILLUMINATE-A Phase 3 Study of Lumasiran, the First Approved Therapeutic for Primary Hyperoxaluria 1
Date of Completion: September 11, 2025
Disclosure Updated Date: September 11, 2025

ASN American Society of Nephrology | JASN Journal of the American Society of Nephrology | KIDNEY360 Accessing Our World From Every Angle | CJASN Clinical Journal of the American Society of Nephrology

ASN Journal Disclosure Form

As per ASN journal policy, I have disclosed any financial relationships or commitments I have held in the past 36 months as included below. I have listed my Current Employer below to indicate there is a relationship requiring disclosure. If no relationship exists, my Current Employer is not listed.

J. Groothoff reports the following:
Employer: Amsterdam UMC, University of Amsterdam; Consultancy: Novo Nordisk; Research Funding: Novo Nordisk; Alnylam;; Honoraria: Alnylam, NovoNordisk; and Advisory or Leadership Role: Alnylam.

I understand that the information above will be published within the journal article, if accepted, and that failure to comply and/or to accurately and completely report the potential financial conflicts of interest could lead to the following: 1) Prior to publication, article rejection, or 2) Post-publication, sanctions ranging from, but not limited to, issuing a correction, reporting the inaccurate information to the authors' institution, banning authors from submitting work to ASN journals for varying lengths of time, and/or retraction of the published work.

Name: Jaap Groothoff
Manuscript ID: CJASN-2025-000806
Manuscript Title: ixty-Month Follow-Up of the ILLUMINATE-A Phase 3 Study of Lumasiran, the First Approved Therapeutic for Primary Hyperoxaluria 1
Date of Completion: September 8, 2025
Disclosure Updated Date: September 8, 2025

ASN
American Society of Nephrology
JASN
Journal of the American Society of Nephrology
KIDNEY360
Accessing Our World From Every Angle
CJASN
Clinical Journal of the American Society of Nephrology

ASN Journal Disclosure Form

As per ASN journal policy, I have disclosed any financial relationships or commitments I have held in the past 36 months as included below. I have listed my Current Employer below to indicate there is a relationship requiring disclosure. If no relationship exists, my Current Employer is not listed.

J. Hogan reports the following:
Employer: Assistance publique Hopitaux de Paris; Emory University; Consultancy: Alnylam, Traverse, Novartis, Biocodex, Recordati Rare Diseases, Roche; and Research Funding: CareDx.

I understand that the information above will be published within the journal article, if accepted, and that failure to comply and/or to accurately and completely report the potential financial conflicts of interest could lead to the following: 1) Prior to publication, article rejection, or 2) Post-publication, sanctions ranging from, but not limited to, issuing a correction, reporting the inaccurate information to the authors' institution, banning authors from submitting work to ASN journals for varying lengths of time, and/or retraction of the published work.

Name: Julien Hogan
Manuscript ID: CJASN-2025-000806
Manuscript Title: Sixty-Month Follow-Up of the ILLUMINATE-A Phase 3 Study of Lumasiran, the First Approved Therapeutic for Primary Hyperoxaluria 1
Date of Completion: August 11, 2025
Disclosure Updated Date: August 11, 2025

ASN American Society of Nephrology
JASN Journal of the American Society of Nephrology
KIDNEY360 Accessing Our World From Every Angle
CJASN Clinical Journal of the American Society of Nephrology

ASN Journal Disclosure Form

As per ASN journal policy, I have disclosed any financial relationships or commitments I have held in the past 36 months as included below. I have listed my Current Employer below to indicate there is a relationship requiring disclosure. If no relationship exists, my Current Employer is not listed.

S. Hulton reports the following:
Employer: Great Ormond Street Hospital for Children, London UK from March 2025; Birmingham Womens' and Childrens' Hospital NHS Foundation Trust, Birmingham UK until February 2025; Consultancy: Alnylam; Arbor Biotechnologies; Research Funding: Alnylam; Honoraria: Alnylam; Chiesi; Advisory or Leadership Role: Trustee UK Kidney Care Charity; Conflict Management Champion for NHS England, UK; Speakers Bureau: Alnylam;; and Other Interests or Relationships: Trustee UK Kidney Care Charity; Conflict Management Champion for NHS England, UK;.

I understand that the information above will be published within the journal article, if accepted, and that failure to comply and/or to accurately and completely report the potential financial conflicts of interest could lead to the following: 1) Prior to publication, article rejection, or 2) Post-publication, sanctions ranging from, but not limited to, issuing a correction, reporting the inaccurate information to the authors' institution, banning authors from submitting work to ASN journals for varying lengths of time, and/or retraction of the published work.

Name: Sally Hulton
Manuscript ID: CJASN-2025-000806
Manuscript Title: Sixty-Month Follow-Up of the ILLUMINATE-A Phase 3 Study of Lumasiran, the First Approved Therapeutic for Primary Hyperoxaluria 1
Date of Completion: September 8, 2025
Disclosure Updated Date: September 8, 2025

ASN American Society of Nephrology JASN Journal of the American Society of Nephrology KIDNEY360 Accessing Our World From Every Angle CJASN Clinical Journal of the American Society of Nephrology

ASN Journal Disclosure Form

As per ASN journal policy, I have disclosed any financial relationships or commitments I have held in the past 36 months as included below. I have listed my Current Employer below to indicate there is a relationship requiring disclosure. If no relationship exists, my Current Employer is not listed.

C. Kaspar reports the following:
Employer: Alnylam Pharmaceuticals; TScan Therapeutics; Nura Bio; Scalera Consulting LLC; Consultancy: Alnylam Pharmaceuticals; TScan Therapeutics; Nura Bio; and Ownership Interest: Alnylam Pharmaceuticals.

I understand that the information above will be published within the journal article, if accepted, and that failure to comply and/or to accurately and completely report the potential financial conflicts of interest could lead to the following: 1) Prior to publication, article rejection, or 2) Post-publication, sanctions ranging from, but not limited to, issuing a correction, reporting the inaccurate information to the authors' institution, banning authors from submitting work to ASN journals for varying lengths of time, and/or retraction of the published work.

Name: Cristin Kaspar
Manuscript ID: CJASN-2025-000806
Manuscript Title: Sixty-Month Follow-Up of the ILLUMINATE-A Phase 3 Study of Lumasiran, the First Approved Therapeutic for Primary Hyperoxaluria 1
Date of Completion: July 16, 2025
Disclosure Updated Date: July 14, 2025

ASN American Society of Nephrology
JASN Journal of the American Society of Nephrology
KIDNEY360 Accessing Our World From Every Angle
CJASN Clinical Journal of the American Society of Nephrology

ASN Journal Disclosure Form

As per ASN journal policy, I have disclosed any financial relationships or commitments I have held in the past 36 months as included below. I have listed my Current Employer below to indicate there is a relationship requiring disclosure. If no relationship exists, my Current Employer is not listed.

J. Lieske reports the following:
Employer: Mayo Clinic; Consultancy: Alnylam; Arbor; Dicerna; OxThera; Allena; Siemens; American Board of Internal Medicine; Lumen; Orfan, Synlogic, Novobiome, Oxidien, Federation Bio, Chinook, BioMarin, Intellia; NovoNordisk; Mirium; Research Funding: Siemens, Alnylam, Dicerna, Siemens, Synlogic, Novobiome; Novo Nordisk, Arbor; Honoraria: American Board of Internal Medicine; American Kidney Fund; Up to Date; Patents or Royalties: Up to Date; and Advisory or Leadership Role: Kidney International, ABIM.

I understand that the information above will be published within the journal article, if accepted, and that failure to comply and/or to accurately and completely report the potential financial conflicts of interest could lead to the following: 1) Prior to publication, article rejection, or 2) Post-publication, sanctions ranging from, but not limited to, issuing a correction, reporting the inaccurate information to the authors' institution, banning authors from submitting work to ASN journals for varying lengths of time, and/or retraction of the published work.

Name: John C. Lieske
Manuscript ID: CJASN-2025-000806
Manuscript Title: Sixty-Month Follow-Up of the ILLUMINATE-A Phase 3 Study of Lumasiran, the First Approved Therapeutic for Primary Hyperoxaluria 1
Date of Completion: August 6, 2025
Disclosure Updated Date: January 13, 2025

ASN
American Society of Nephrology
JASN
Journal of the American Society of Nephrology
KIDNEY360
Accessing Our World From Every Angle
CJASN
Clinical Journal of the American Society of Nephrology

ASN Journal Disclosure Form

As per ASN journal policy, I have disclosed any financial relationships or commitments I have held in the past 36 months as included below. I have listed my Current Employer below to indicate there is a relationship requiring disclosure. If no relationship exists, my Current Employer is not listed.

J. Saland reports the following:
Employer: The Mount Sinai Medical Center; Consultancy: Alnylam Pharmaceuticals, Novo Nordisk; Research Funding: Alnylam Pharmaceuticals; and Honoraria: Alnylam Pharmaceuticals.

I understand that the information above will be published within the journal article, if accepted, and that failure to comply and/or to accurately and completely report the potential financial conflicts of interest could lead to the following: 1) Prior to publication, article rejection, or 2) Post-publication, sanctions ranging from, but not limited to, issuing a correction, reporting the inaccurate information to the authors' institution, banning authors from submitting work to ASN journals for varying lengths of time, and/or retraction of the published work.

Name: Jeffrey Saland
Manuscript ID: CJASN-2025-000806
Manuscript Title: Sixty-Month Follow-Up of the ILLUMINATE-A Phase 3 Study of Lumasiran, the First Approved Therapeutic for Primary Hyperoxaluria 1
Date of Completion: August 19, 2025
Disclosure Updated Date: August 19, 2025

ASN American Society of Nephrology | JASN Journal of the American Society of Nephrology | KIDNEY360 Accessing Our World From Every Angle | CJASN Clinical Journal of the American Society of Nephrology

ASN Journal Disclosure Form

As per ASN journal policy, I have disclosed any financial relationships or commitments I have held in the past 36 months as included below. I have listed my Current Employer below to indicate there is a relationship requiring disclosure. If no relationship exists, my Current Employer is not listed.

A. Sellier-Leclerc reports the following:
Employer: hospices civils de Lyon; Consultancy: ALNYLAM; ALEXION, NOVARTIS, SOBI, BMS; Ownership Interest: ALNYLAM; ALEXION, NOVARTIS, SOBI, BMS; Research Funding: Alnylam; Dicerna Novo Nordisk; BMS; Roche; and Advisory or Leadership Role: Alnylam; ALEXION, Novartis.

I understand that the information above will be published within the journal article, if accepted, and that failure to comply and/or to accurately and completely report the potential financial conflicts of interest could lead to the following: 1) Prior to publication, article rejection, or 2) Post-publication, sanctions ranging from, but not limited to, issuing a correction, reporting the inaccurate information to the authors' institution, banning authors from submitting work to ASN journals for varying lengths of time, and/or retraction of the published work.

Name: Anne-Laure All Sellier-Leclerc
Manuscript ID: CJASN-2025-000806
Manuscript Title: Sixty-Month Follow-Up of the ILLUMINATE-A Phase 3 Study of Lumasiran, the First Approved Therapeutic for Primary Hyperoxaluria 1
Date of Completion: August 10, 2025
Disclosure Updated Date: July 31, 2025
